# Effects of famotidine use during pregnancy: an observational cohort study

**DOI:** 10.1186/s40780-024-00393-3

**Published:** 2024-11-08

**Authors:** Ayako Nishimura, Ayako Furugen, Masaki Kobayashi, Yoh Takekuma, Naho Yakuwa, Mikako Goto, Masahiro Hayashi, Atsuko Murashima, Mitsuru Sugawara

**Affiliations:** 1 Department of Pharmacy, Hokkaido University Hospital, Sapporo, Japan; 2 Laboratory of Clinical Pharmaceutics & Therapeutics, Division of Pharma Sciences, Faculty of Pharmaceutical Sciences, Hokkaido University, Sapporo, Japan; 3 The Japan Drug Information Institute in Pregnancy, National Center for Child Health and Development, Setagaya-Ku, Tokyo, Japan; 4 Department of Pharmacy, Toranomon Hospital, Minato-Ku, Tokyo, Japan; 5 Laboratory of Pharmacokinetics, Faculty of Pharmaceutical Sciences, Hokkaido University, Sapporo, Japan

**Keywords:** Famotidine, Observational cohort study, Pregnancy outcomes, Teratogenicity

## Abstract

**Background:**

Famotidine, a histamine2-receptor antagonist (H2Ras), is widely used to treat and prevent gastrointestinal symptoms during pregnancy. Although several studies have reported the use of H2Ras during pregnancy, limited data on famotidine were included in these reports. Therefore, we analyzed pregnancy outcome data to evaluate the effects of famotidine use during pregnancy on the fetus.

**Methods:**

Pregnancy outcome data were used for females enrolled in two Japanese facilities that provided counseling on drug use during pregnancy between April 1988 and December 2017. For the primary endpoint, the incidence of congenital malformations was calculated from the data of live birth to pregnant women who took famotidine (*n* = 330) or drugs considered to exert no teratogenic risk (control, *n* = 1,407) during the first trimester of pregnancy. Considering secondary endpoints, the incidence of obstetric outcomes, including preterm delivery, was calculated from data on the use of famotidine (*n* = 347) and controls (*n* = 1,476) during the entire pregnancy. The crude odds ratios (cORs) for the incidence of congenital malformations were calculated using univariate logistic regression analysis, with the control group used as the reference. Adjusted ORs (aORs) were calculated using multivariate logistic regression analysis adjusted for various other factors.

**Results:**

The incidences of congenital malformations in the famotidine and control groups were 3.9% and 2.8%, respectively. There was no significant difference between the famotidine and control groups (cOR: 1.40 [95% CI:0.68–2.71], aOR: 1.06 [95% CI:0.51–2.16]). Conversely, the preterm delivery rates were 8.1% and 3.8% in the famotidine and control groups, respectively, indicating a significant difference (cOR: 2.00 [95% CI:1.20–3.27]). However, the multivariate analysis eliminated famotidine use as a confounding factor.

**Conclusions:**

This observational cohort study revealed that exposure to famotidine during the first trimester of pregnancy was not associated with an increased risk of congenital malformations in infants. Although a higher rate of preterm delivery was detected in famotidine users when compared with controls, this could be attributed to confounding factors, such as complications.

## Background

Famotidine, a histamine2-receptor antagonist (H2Ra), is widely used to treat and prevent gastrointestinal symptoms. In pregnant women, famotidine is prescribed to prevent the side effects of drugs used to treat complications and manage gastrointestinal symptoms specific to pregnant women.

Heartburn and symptomatic gastroesophageal reflux disease are common clinical symptoms that occur during pregnancy [1–6]. Heartburns are present in 30–50% of pregnant women, often as high as 80%. Approximately 17% of pregnant women experience both heartburn and reflux symptoms [3–5]. Typically, these symptoms begin during the first trimester of pregnancy, although studies have documented reflux symptoms in nearly 25% of pregnant women throughout all trimesters of pregnancy [3]. The severity of heartburn reportedly increases during pregnancy. During pregnancy, the occurrence of symptoms can be attributed to fluctuations in sex hormone levels and uterine enlargement, which functionally and physically affect gastrointestinal motility. Because heartburn symptoms are more common but less severe, lifestyle changes are initially recommended, including improving the meals consumed. If there is no adequate response or severe symptoms are present, pharmacotherapy is initiated initially with antacids, followed by H2Ras or proton pump inhibitors [6].

Several studies have reported the use of H2Ras during pregnancy. However, these reports mainly contain data on ranitidine and cimetidine, and the number of famotidine users included in these reports is small when compared with those taking other H2Ras [7–12]. Ranitidine, on the other hand, has recently disappeared from the market due to concerns that N-nitroso dimethylamine is present as an impurity above acceptable levels. Studies on famotidine and cimetidine have not revealed similar concerns [13]. In addition, cimetidine has been reported to strongly inhibit hepatic drug-metabolizing enzymes P-450, particularly CYP3A4 and CYP2D6, which limits its use due to potential interactions concomitant medications. Famotidine has no effect on hepatic drug-metabolizing enzymes; however, dosage adjustments are necessary based on renal function. Additionally, in animal studies, cimetidine has been reported to have anti-androgenic effects, whereas famotidine does not [14]. Famotidine is marketed as an over-the-counter (OTC) medication and is commonly used by women who want to get pregnant due to its safety [15]. Therefore, we analyzed pregnancy outcome data of females enrolled at two Japanese facilities to evaluate the effects of famotidine use during pregnancy on the fetus.

## Materials and methods

### Data collection

A combined database of pregnancy outcomes was prepared by extracting data from the clinical databases of two Japanese facilities that provide counseling on drug use during pregnancy, including the Counseling Clinic for “Pregnancy and Medicine” of Toranomon Hospital. Data from female subjects who consulted the counseling clinic between April 1988 and December 2016 were included in the study. The second center was the Japan Drug Information Institute in Pregnancy, the National Center for Child Health and Development, and data from females who sought consultation regarding the safety of drug use during pregnancy between October 2005 and December 2017 were included. Pregnancy outcomes and neonatal data were collected through correspondence or telephone one to several months after the expected delivery date. Major malformations were defined according to the European Surveillance of Congenital Anomalies (EUROCAT) [16]. If congenital anomalies were not included in EUROCAT, a diagnosis was made by a congenital anomaly specialist.

**Fig. 1:**
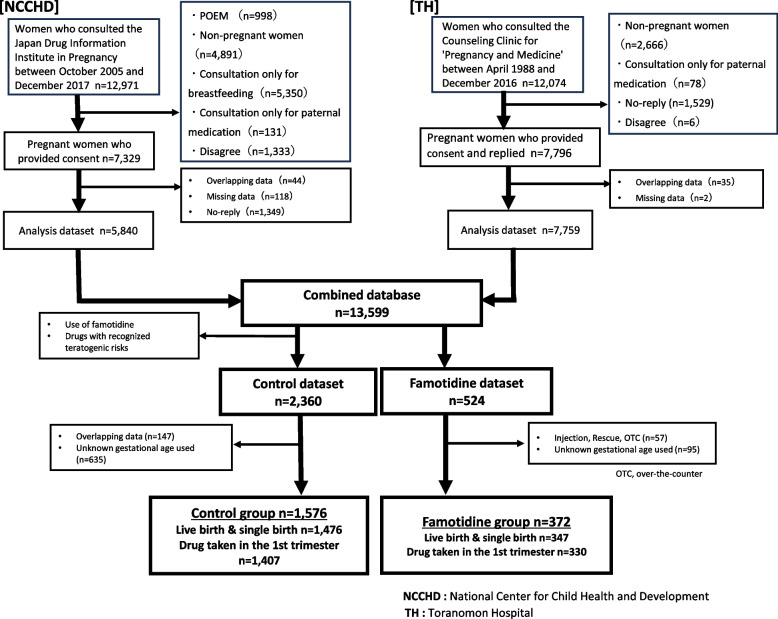
Flowchart for dataset fixation

Patients using famotidine were excluded, and those using drugs considered to exert no teratogenic risk were extracted from the combined database to create a control dataset. A control group (*n* = 1,576) was derived from this dataset by excluding duplicate data and cases without information on the period of pregnancy when the drug was used. Information regarding the gestational period of drug exposure is critical when discussing the effects of drug exposure on the fetus during pregnancy. Therefore, cases missing this information were excluded from the analysis. The analysis used 1,476 cases, excluding abortions, miscarriages, stillbirths, and multiple births. Patients using famotidine were extracted from the combined database to form a famotidine dataset. Cases that involved the use of famotidine injection, use of abortive medications, and over-the-counter drug use were excluded from this dataset. Injectable famotidine was excluded because the background of patients who needed injectable famotidine was likely to markedly differ from that of patients in the control group, most of whom administered oral famotidine. We excluded patients who took abortive and over-the-counter medications due to unclear indications for their use, and only included patients with regular oral intake in the analysis. Additionally, as with the control group, those without information on gestational use were excluded from the famotidine group (*n* = 372). After excluding abortions, miscarriages, stillbirths, and multiple births, 347 cases were included in the final analysis (Fig. 1).

### Study endpoint and statistical analysis

The primary endpoint of this study was the incidence of major malformations. To analyze the risk of congenital anomalies, cases in which the drug was used during the first trimester were included in the famotidine and control groups. Secondary endpoints were delivery outcomes (birth weight, weeks at birth, and preterm birth rate). Cases in which prescribed drugs were used during any gestational period were included in the analysis.

Data were analyzed using the statistical analysis software R (version 4.2.2). Statistical comparisons between the famotidine group and the control group were performed using the Fisher exact test for binary variables such as malformation incidence and the Mann–Whitney U test for continuous variables such as maternal age and birth weight. Statistical significance was set at *P* < 0.05.

Crude odds ratios (cORs) for the incidence of malformations and preterm birth were calculated using univariate logistic regression analysis, using the control group as the reference. Adjusted ORs (aORs) were calculated using multivariate logistic regression analysis adjusting for maternal age, alcohol consumption, smoking habits, pregnancy history, and delivery history. These confounding factors are often used in reports discussing outcomes related to drug exposure during pregnancy [11, 12]. These are also factors that have been shown to contribute to the risk of fetal abnormalities and　obstetric -complications leading to preterm delivery [17–20]. The confidence interval (CI) was set at 95%.

### Ethics statement

This study was approved by the Ethics Committees of the National Center for Child Health and Development, Toranomon Hospital and Hokkaido University Hospital. The study was conducted in accordance with the principles of the Declaration of Helsinki. Informed consent was obtained from all participants. The collected information was entered into a database and de-identified by an information manager. As a result, none of the individuals were identified by the investigators.

## Results

### Maternal background and pregnancy outcomes

Table 1 presents the maternal background of the famotidine group (*n* = 372) and control group (*n* = 1,576). Drug use during the first trimester was higher in both groups. There were no statistically significant differences in the maternal age distribution. The percentage of females aged over 35 years was approximately 20% in both groups. Alcohol consumption during pregnancy was observed in 26.1% and 23.6% of females in the famotidine and control groups, respectively. Smoking during pregnancy was 7.8% and 11.9% in the famotidine and control groups, respectively. There were no significant differences between the two groups. With regard to pregnancy history, 59.3% of the famotidine group had no history of pregnancy, whereas 59.0% of the control group had a history of pregnancy. Considering delivery history, 66.1% of the females in the famotidine group had no history of delivery, whereas the number of females in the control group with or without a history of delivery was almost equal.

**Table 1:** Patient characteristics

		Famotidine group	Control group
*Total, n*		372	1,576
*Use period, n*	1st trimester	348	1,505
	2nd,3rd trimester	18	70
	All trimesters	6	1
*Age(year), n(%)*	50% [25%,75%]	31 [27, 34]	30 [27, 34]
	≧35	87 (23.4)	320 (20.3)
	< 30	285 (76.6)	1,256 (79.7)
*Alcohol, n(%)*	Use related to pregnancy	97 (26.1)	372 (23.6)
	Use unrelated to pregnancy	259 (69.6)	1,018 (64.6)
	NA	16 (4.3)	186 (11.8)
*Smoking, n(%)*	Use related to pregnancy	47 (7.8)	188 (11.9)
	Use unrelated to pregnancy	309(88.2)	1,240 (78.7)
	NA	16(4.0)	148 (9.4)
*Pregnancy history, n(%)*	History of pregnancy	177 (40.7)	930 (59.0)
	No prior pregnancy	195 (59.3)	638 (40.5)
	NA	0	8(0.5)
*Delivery history, n(%)*	History of delivery	126 (33.9)	792 (50.3)
	No prior delivery	246 (66.1)	774 (49.1)
	NA	0	10 (0.6)

*NA* Not available

Table 2 summarizes the pregnancy results. There were no differences in stillbirth or miscarriage rates between the two groups. The famotidine-treated group had a slightly higher abortion rate than the control group.

**Table 2:** Pregnancy outcomes

	Famotidine group	Control group
*n*	372	1,576
*Outcomes, n(%)*		
*Live birth*	351 (94.4)	1,488(94.4)
*Single birth*	347 (98.9^a^)	1,476(99.2^a^)
*Multiple births*	1 (0.3^a^)	5(0.3^a^)
*NA*	3 (0.9^a^)	7(0.5^a^)
*Stillbirth*	0 (0.0)	4 (0.3)
*Miscarriage*	14 (3.8)	70 (4.4)
*Abortion*	7 (1.9)	13 (0.8)
*Other*	0 (0.0)	1 (0.1)

Stillbirth: Fetal death after 22 weeks gestation

*NA* not available

^a^% in the Live birth

### Risk of congenital malformations

The incidence of all congenital malformations, including minor malformations, was 3.9% and 2.8% in the famotidine and control groups, respectively, with no statistically significant difference observed. The cOR was 1.40 [95%CI: 0.68–2.71], and the aOR for maternal age, smoking, alcohol use, pregnancy, and delivery history was 1.06 [95%CI: 0.51–2.16]. The incidence of major malformations was 3.3% in the famotidine group and 1.9% in the control group, with a cOR of 1.76 [95%CI: 0.78–3.72] and an aOR of 1.26 [95%CI: 0.56–2.86], showing no significant increase in incidence in the famotidine group (Table 3). Congenital malformations observed in the famotidine group included cardiac malformations, synovial encephalopathy, scrotal edema, sacral depression, clubfoot, duplicated vagina, cleft lip and palate, and otorhinostomies, with no consistent trends detected (Table 4).

**Table 3:** Rate of malformation

	Congenital malformation						
	Yes	No	Expression rate (%)	Crude OR [95%CI]	*P*-value	Adjusted OR [95%CI]	*P* value
Control group (*n* = 1407)	40	1,367	2.8	1	-	1	-
Famotidine group (*n* = 330)	13	317	3.9	1.40 [0.68–2.71]	0.288	1.06 [0.51**–**2.16]	0.883
	Major malformation						
	Yes	No	Expression rate (%)	Crude OR [95%CI]	*P* value	Adjusted OR [95%CI]	*P* value
Control group (*n* = 1,407)	27	1,380	1.9	1	-	1	-
Famotidine group (*n* = 330)	11	319	3.3	1.76 [0.78–3.72]	0.288	1.26 [0.56–2.86]	0.579

*CI* confidence interval, *OR* odds ratio

**Table 4:** Type of congenital malformation

	Famotidine group (*n* = 330)	Control group (*n* = 1,407)
*Congenital heart disease n,(%)*	5(1.5)	16(1.1)
*Endocardial cushion defect (ECD)*	1	1
*Ventricular septal defect (VSD)*	3	5
*VSD* + *Atrial septal defect (ASD)*	1	1
*VSD* + *Plumonary stenosis (PS)* + *Zygodactyly*		1
*VSD* + *Aortic stenosis (AS)*		1
*PS* + *Single ventricle*		1
*PS*		1
*Patent ductus arteriosus (PDA)*		1
*Patent foramen ovale (PFO)*		1
*Tetralogy of Fallot*		1
*Right ventricular initiation of taelar vessels*		1
*Complete transposition of great arteries*		1
*Lissencephaly*	1	
*Esophageal atresia*		1
*Cleft lip and palate*	1	1
*Tongue adhesion*		1
*Esotropia*		1
*Polydactylia*		3
*Aural fistula*	2	
*Sacral dimple*	1	
*Inversion of foot*	1	1
*Strawberry mark*		1
*Birthmark*		2
*Dermal sinus*		1
*Cystic disease of kidney*		1
*Hydronephrosis*		2
*Enlargement of renal pelvis*		1
*Double vaginas*	1	
*Scrotal hydrops*	1	1
*Adhesion of scrotum and penis*		1
*Cryptorchid*		1
*Atresia of anus*		1
*Inguinal hernia*		1
*Hydrops fetalis*		1
*Down’s syndrome, rectus muscle separation*		1
*Congenital hypothyroidism*		1
***Total***	**13**	**40**

### Neonatal data and risk of preterm birth

Although the two groups did not differ in the median number of weeks of gestation at delivery, there was a significant difference in the incidence of preterm delivery at less than 37 weeks of gestation (8.1% in the famotidine group and 3.8% in the control group). The incidence of preterm delivery at an earlier gestational age (< 34 weeks) was higher in the famotidine group than in the control group. The median birth weight was 2,942 g and 3,050 g in the famotidine and control groups, respectively; however, this difference was not clinically significant. Nevertheless, 13.3% of infants in the famotidine group had a low birth weight, and 1.4% had a very low birth weight, indicating the famotidine group had a higher proportion of infants with low small birth weights than the control group (Table 5).

**Table 5:** Preterm birth rate and maternal diseases in preterm cases

	Famotidine group (*n* = 347)	Control group (*n* = 1,476)	*P* value
*Birth weeks Median [25%,75%],weeks*	39 [38,40]	39 [38,40]	
*Preterm birth (*< *37 weeks), n(%)*	28 (8.1)	56 (3.8)	*P* < 0.01
< *34 weeks, n(%)*	11(3.2)	10 (0.7)	*P* < 0.01
*Birth weight Median [25%,75%], g*	2,942 [2,725, 3,223]	3,050 [2,805, 3,300]	
< *2500 g, n(%)*	46 (13.3)	101 (6.8)	*P* < 0.01
< *1500 g, n(%)*	5 (1.4)	6 (0.4)	*P* < 0.05
*Maternal diseases in preterm cases, n*			
*Still’s disease*	1		
*Systemic lupus erythematosus (SLE)*	6		
*Rheumatoid arthritis (RA)*	1		
*Antiphospholipid-antibody syndrome (APS)*	1		
*Ulcerative colitis(UC)*	1		
*Thyroid disease*	1	1	
*Allergies*	1	4	
*Asthma*	2	1	
*Marfan’s syndrome*	1		
*pituitary adenoma*	1		
*Myasthenia gravis*	1		
*Parkinson’s disease*	1		
*Hepatitis C*		1	
*Ventricular septal defects(VSD)*	1		
*Tooth decay*	1		
*Skin diseases*		4	
*Uterine fibroid*		1	
*Gastroenteritis*		1	
*History of tuberculosis*		2	
*Chronic fatigue syndrome*		1	

The primary conditions responsible for preterm delivery were identified in both groups. The famotidine group was associated with a high prevalence of inflammatory autoimmune diseases, such as systemic lupus erythematosus, antiphospholipid antibody syndrome, and ulcerative colitis. Therefore, univariate and multivariate analyses were performed, with maternal inflammatory autoimmune diseases (Still syndrome, systemic lupus erythematosus, antiphospholipid antibody syndrome, rheumatoid arthritis, and ulcerative colitis) as confounding factors. The results showed that the cOR for famotidine use was 2.00 [95%CI: 1.20–3.27], and the cOR for complications of inflammatory autoimmune diseases was 6.35 [95%CI: 2.24–17.19]. Multivariate analysis of statistically significant factors excluded famotidine as a confounding factor (Table 6).

**Table 6:** Analysis of causes of preterm birth

	OR [95%CI]	*P* value
*Univariate analysis*		
* Age*		
< *35 years*	1.00	
*≧ 35 years*	1.44 [0.84,2.40]	0.169
* Alchol habit*		
*No*	1.00	
*Yes*	0.78 [0.41,1.38]	0.424
* Smoke habit*		
*No*	1.00	
*Yes*	0.94 [0.33,2.21]	1
* Pregnancy history*		
*No*	1.00	
*Yes*	0.48 [0.29,0.76]	0.00141
* Delivery history*		
*No*	1.00	
*Yes*	0.49 [0.29,0.80]	0.00329
* Autoimmune inflammatory disease*		
*No*	1.00	
*Yes*	6.35 [2.24,17.19]	0.00026
* Famotidine use*		
*No*	1.00	
*Yes*	2.00 [1.20,3.27]	0.0053
*Multivariate analysis*		
* Pregnancy history*		
*No*	1.00	
*Yes*	0.15 [0.06,0.42]	0.00025
* Autoimmune inflammatory disease*		
*No*	1.00	
*Yes*	6.08 [2.30,16.10]	0.00028

*CI* Confidence interval, *OR* Odds ratio

## Discussion

The use of H2Ras during pregnancy has been extensively documented [7–12]. In a 2009 meta-analysis, data from 2,398 H2Ras-exposed and 119,892 non-exposed subjects found no increase in the risk of teratogenicity (OR: 1.14 [95%CI: 0.45–1.45]) [9]. Based on these data, H2Ras could be used to treat heartburn and gastric acid reflux in pregnant women. However, previous reports provide limited information on H2Ras use, particularly individual agents. In animals, no teratogenic or reproductive changes have been observed at famotidine doses significantly higher than clinical doses, and no contraindications to human administration have been documented [21]. A 1996 prospective cohort study conducted by the Motherisk Program, a teratology information service in Toronto, Canada, compared 178 H2Ras-exposed pregnancies (8% of whom were exposed to famotidine) with 178 unexposed patients and detected major malformation rates of 2.1% and 3.5%, respectively [10]. In 2005, a report by the European Network of Teratology Information Services (ENTIS) examined 553 H2Ras-exposed pregnancies (75 of whom were exposed to famotidine) with controls. The incidence of major malformations was 2.7% in the H2Ras group and 3.5% in the control group (risk ratio [RR], 0.78 [95%CI: 0.42–1.44]) [11]. In 2010, an analysis of babies born to women with early pregnancy exposure to H2Ras was conducted using the database of Israel health maintenance organization registry. The authors identified 878 cases of famotidine exposure, and the incidence of major congenital malformations was 6.6% in the famotidine group and 5.2% in the non-exposed group (aOR: 1.21[95%CI: 0.92–1.58]), which was not significantly increased when compared with that in the non-exposed group [12]. Although this study included a large number of subjects, it involved the analysis of a prescription data and did not confirm its actual use. To the best of our knowledge, our study is the largest prospective study on the safety of famotidine use during pregnancy. Herein, we found that the overall incidence of congenital malformations in the famotidine group was 3.9%, and the incidence of major malformations was 3.3%, which did not exceed the baseline risk (3–5%). Our findings are consistent with the frequency of congenital malformations reported in the latest Japanese branch of the International Clearinghouse for Birth Defects Surveillance and Research [22]. The aORs of the control group were similar to those reported previously for overall congenital malformations (aOR: 1.06 [95%CI: 0.51–2.16]) and major malformations (aOR: 1.26 [95%CI: 0.56–2.86]). No specific trend was observed in the occurrence of malformations in the famotidine group. Although it is impossible to establish a precise conclusion based on the insufficient number of subjects included in the current study, the 1.5% incidence of cardiac malformations is consistent with that reported by the Neonatal Congenital Heart Disease Surveillance Report [23] and the Pediatric Heart Disease Study [24]. Importantly, exposure to famotidine during early pregnancy did not result in an increase in the incidence of specific malformations. To date, there have been no reports of H2Ras exposure during pregnancy indicating an increase in certain malformations such as cardiac malformations, and the results of this study are consistent with these reports [11, 12]. These findings suggest that exposure to famotidine during the first trimester of pregnancy does not increase the risk of developing congenital malformations.

Neonatal data revealed higher rates of preterm delivery and low birth weight in the famotidine group than in the control group. Although these rates are high when compared with the global average [25, 26], they are higher than those reported in recent Japanese studies [27, 28], which reported preterm birth rates of 4.6%, low birth weight rate of 9.4%, and very low birth weight rate of 0.7%. Although ENTIS has reported a higher preterm birth rate (RR: 1.67 [95%CI: 1.18–2.35]) in the H2Ras group, the reason for this remains unclear [11]. Conversely, Ilan et al. in 2010 reported no significant differences in the preterm birth rate or percentage of low and very low birth weight infants between the H2Ras and control groups [12].

Therefore, we focused on the maternal complications of preterm delivery and found that the famotidine group included pregnant women with autoimmune inflammatory disease complications. Autoimmune inflammatory diseases have been associated with preterm delivery [29–33]. The results of the multivariate analysis suggest that the effect of famotidine use during pregnancy may be minimal, and that the presence of an autoimmune inflammatory disease could be an influencing factors. However, no definitive conclusions can be drawn from this analysis alone. The use of steroids to treat autoimmune inflammatory diseases may be a risk factor for preterm delivery [29, 31]. In the famotidine group in the current study, concomitant steroid use during pregnancy was detected in 17% of patients, and the rate of use in the preterm group (47%) was higher than that in the full-term delivery group (16%). This may be partly due to the oral use of famotidine as a prophylactic agent to address steroid-related side effects, which may have impacted our findings.

Given that the current study is based on the clinical databases of two Japanese institutions providing counseling on drug use during pregnancy, the information was obtained during interviews conducted at a single time point by the counselors themselves and not from direct observation throughout the pregnancy period. Therefore, there is a lack of detailed information on patient backgrounds, presence or absence of obstetric complications after consultation, and status of medications taken. Recently, an association between the use of antacids, such as H2Ras, during pregnancy and asthma and allergic symptoms in children was reported [34, 35]. However, after examining confounding factors by indication and familial factors, some reports showed a negative association with the use during pregnancy [36]. The limitations of our analysis of the association between famotidine and preterm delivery include the lack of accounting for confounding factors such as concomitant medications and family factors. We considered the presence of an autoimmune inflammatory disease in the mother as one of the factors, but the presence of this complication may have influenced the presence of steroids and non-steroidal concomitant medications, and the presence of these concomitant medications may have resulted in an interaction with the use of famotidine. Therefore, we believe that further analysis, including stratified analysis in the presence or absence of autoimmune disease, is needed to clarify whether famotidine is involved, and this is a topic for future studies. Furthermore, pregnancy outcomes were determined via interview approximately one month postpartum; hence, the long-term effects of famotidine on the growth and development of children, including its association with asthma and allergic symptoms in children, warrant further investigation in future research.

## Conclusions

Collectively, our findings suggest that famotidine exposure during the first trimester of pregnancy does not increase the risk of congenital malformations. Exposure to famotidine during the entire gestational period did not appear to impact neonatal abnormalities or pregnancy outcomes, although further investigations of its association with preterm delivery are warranted.

## Data Availability

The datasets used and/or analyzed during the current study are available from the corresponding author on reasonable request.

## Abbreviations

H2Ras: Histamine2-receptor antagonist
cORs: Crude odds ratios
aORs: Adjusted odds ratios
EUROCAT: The European Surveillance of Congenital Anomalies
ENTIS: The European Network of Teratology Information Services
